# Different Rates of Bioprosthetic Aortic Valve Failure With Perimount™ and Trifecta™ Bioprostheses

**DOI:** 10.3389/fcvm.2021.822893

**Published:** 2022-01-20

**Authors:** Rüdiger Lange, Zahra Alalawi, Stephanie Voss, Johannes Boehm, Markus Krane, Keti Vitanova

**Affiliations:** ^1^Department of Cardiovascular Surgery, German Heart Center Munich, Technische Universität München, Munich, Germany; ^2^Insure (Institute of Translational Cardiac Surgery), Department of Cardiovascular Surgery, German Heart Center Munich, Technische Universität München, Munich, Germany; ^3^DZHK (German Center for Cardiovascular Research)–Partner Site Munich Heart Alliance, Munich, Germany; ^4^Division of Cardiac Surgery, Department of Surgery, Yale University School of Medicine, New Haven, CT, United States

**Keywords:** BVF, SAVR-surgical aortic valve replacement, bioprostheses, bioprosthesis adverse effects, bioprosthesis avr

## Abstract

**Objectives:**

The use of bioprostheses in surgical aortic valve replacement (SAVR) has increased in younger patients. Comparative analysis of different types of bioprostheses is lacking. We aimed to compare two proprietary bioprostheses with different designs, i.e., internally and externally mounted leaflets, focusing on the long-term durability and survival.

**Methods:**

We conducted a large single-center retrospective analysis of all consecutive patients who underwent SAVR with either Perimount™ or Trifecta™ bioprostheses between 2001 and 2019. The patient groups were further subdivided by age <65 and >65. Endpoints of the study were all-cause mortality and reoperation due to bioprosthetic valve failure (BVF).

**Results:**

Selection criteria resulted in a total sample of 5,053 patients; 2,630 received a Perimount prosthesis (internally mounted leaflets) and 2,423 received a Trifecta prosthesis (externally mounted leaflets). The mean age at surgery was similar (69 ± 11 y, PM, and 68 ± 10 y, TF, *p* = 0.9), as was estimated survival at 8 years (76.1 ± 1.3%, PM, and 63.7 ± 1.9% TF; p=0.133). Patients in the Trifecta group had a significantly higher cumulative reoperation rate at 8 years compared to those in the Perimount group (16.9 ± 1.9% vs. 3.8 ± 0.4%; *p* < 0.01). This difference persisted across age groups (<65 y, 13.3% TF vs. 8.6% PM; >65 y, 12% TF vs. 7% PM).

**Conclusion:**

Bioprostheses for SAVR with externally mounted leaflets (Trifecta) showed significantly higher long-term reoperation rates compared to those with internally mounted leaflets (Perimount), regardless of the patient's age at SAVR. Survival was similar with both bioprostheses.

## Introduction

Surgical implantation of bioprosthetic aortic valves has become more common in patients under the age of 65. As such, a comparative analysis of the long-term durability and the clinical outcomes of these valves is gaining increasing clinical importance. Navigating the relevant literature reveals controversial data regarding the durability and the hemodynamic effect of different designs of prosthetic valves. Prostheses with externally mounted leaflets may translate into larger effective orifice areas, which favors improved hemodynamics. In contrast, a prosthesis design with leaflets mounted inside the stent may offer greater stent flexibility to absorb energy and reduce leaflet stress.

In the present study, two bioprostheses for surgical aortic valve replacement (SAVR) with differing design, the Edwards Perimount™ (PM; Carpentier-Edwards [CE] Perimount; Edwards Lifesciences, Irvine, CA) and the Abbott St. Jude Trifecta™ (TF; Abbott Structural Heart, St Paul, MN) were investigated. The Perimount pericardial bioprosthesis is a trileaflet valve designed for supra-annular implantation and consisting of bovine pericardial leaflets mounted internally underneath a flexible cobalt-chromium stent. The Trifecta valve is a trileaflet stented valve, also designed for supra-annular placement, which consists of valve leaflets manufactured from a single bovine pericardial tissue strip that is externally mounted onto the titanium alloy stent frame, which is covered with porcine pericardial tissue allowing for only tissue-to-tissue contact during valve function.

The objective of this retrospective, observational study is to present a comparative analysis of the two different valve designs of these devices, with a particular focus on durability.

## Materials and Methods

### Study Design

This is a large, single-center, retrospective analysis of all consecutive patients who underwent surgical aortic valve replacement (SAVR) using Perimount or Trifecta bioprostheses at the German Heart Centre, Munich, between 2001 and 2019. Exclusion criteria were concomitant aortic root procedures for acute type A aortic dissection (ATAAD), aortic valve endocarditis, SAVR with mechanical prosthesis or other bioprosthesis. Patient data were identified from our internal clinical database. All medical reports including operative protocols and in-hospital and outpatient notes were reviewed. Selection criteria resulted in a total sample of 5,053 patients, 2,630 with Perimount prostheses and 2,423 with Trifecta prostheses.

The study was approved by the Institutional Review Board of the Technical University of Munich (129/21 S from March/5/2021). The approval included a waiver of informed patient consent.

Endpoints of the study were all-cause mortality and reoperation due to bioprosthetic valve failure (BVF), as defined according to the Consensus on Bioprosthetic Valve Deterioration (1). The decision to perform either Redo-SAVR or ViV TAVR was made in an interdisciplinary insitutional Heart-Team, weighing individualized patient-tailored operative risk. Follow-up was completed for 91% of the cohort. All patients received anticoagulants (phenprocoumon) at discharge for at least 3 months, or for a lifelong regimen if there were other indications.

### Statistical Analysis

All statistical analyses were performed using the Statistical Package for the Social Sciences (SPSS), version 24.0 for Windows (IBM, Ehningen, Germany), the R-Project for Statistical Computing and Data Science, and the Number Cruncher Statistical System (NCSS) Data Analysis Software. Categorical variables were presented as absolute numbers and percentages. A Chi-square test (Fisher's correction test) was used for categorical data between groups. Continuous variables were expressed as means ± standard deviations or medians with minimum and maximum ranges, as appropriate. An independent sample *t* test was used to compare groups with normally distributed variables and the Mann-Whitney test was used for variables that were not normally distributed. Kaplan-Meier survival curves were computed to present the endpoints. Differences in the endpoints were evaluated using the log-rank Mantel Cox test. In addition, a competing risk analysis was performed in order to correctly estimate marginal probability of an event in the presence of competing events. A Cumulative Incidence Function (CIF) was used to solve this particular issue by estimating the marginal probability of a certain event as a function of its cause-specific probability and overall survival probability. The competing risk analysis included a non-parametric method which involves the use of a modified Chi-squared test to compare CIF curves between groups, and a parametric approach which model the CIF based on a subdistribution hazard ratios (HRs) with 95% confidence interval, i.e., Gray test. *P*-values ≤ 0.05 were considered statistically significant.

## Results

### Patients

The pre-operative patient characteristics are presented in Table 1. The mean age at surgery was 69 ± 11 y [median 71, (20 to 91), IQR = 65–77.6] in the Perimount group and 68 ± 10 y [median 71 (22 to 94), IQR = 62–75] in the Trifecta group (*p* = 0.9). Chronic obstructive pulmonary disease (COPD) and pulmonary hypertension (PHT) were more common at time of surgery in the Trifecta group, while patients in the Perimount group had a higher degree of physical impairment (NYHA III) at time of surgery.

**Table 1:** Baseline characteristics of patients receiving surgical aortic valve replacement (SAVR) with Perimount or Trifecta bioprostheses.

	**Perimount** ***n*** **= 2,630**	**Trifecta** ***n*** **= 2,423**	***p*** **-value**
Age, years	69 ± 11	68 ± 10	0.9
Sex, men, *n* (%)	1,499 (57)	1,429 (59)	0.8
Height, cm	167 ± 19	171 ± 10	0.8
Weight, kg	61 ± 20	61 ± 14	0.8
Chronic obstructive pulmonary disease, *n* (%)	319 (12)	471 (19)	**<0.01**
Peripheral vascular disease, *n* (%)	162 (2)	147 (6)	0.91
Hyperlipoproteinemia, *n* (%)	2,483 (94)	2,324 (96)	0.78
Arterial hypertension, *n* (%)	2,551 (97.6)	2,375 (98)	0.65
Pulmonary hypertension, *n* (%)	341 (13.5)	533 (21.8)	**<0.01**
Diabetes mellitus-NIDDM, *n* (%)	2,025 (77)	1,865 (77)	0.42
Renal failure, *n* (%)	39 (1.5)	36 (1.5)	0.9
NYHA III	1,656 (62.9)	1,211 (57.5)	**<0.01**

*Significant values are bold*.

The complexity of the surgery was balanced between both groups, with 2,130 and 1,986 patients receiving isolated SAVR with Perimount and Trifecta, respectively, and 500 patients with Perimount and 437 with Trifecta undergoing SAVR with concomitant cardiac procedure. Operative details are given in Table 2. Aortic stenosis was the predominant indication for SAVR in both groups; aortic insufficiency was more frequent in the Trifecta group (*p* < 0.01, Table 2).

**Table 2:** Operative data in patients after surgical aortic valve replacement (SAVR) with Perimount or Trifecta bioprosthesis.

	**Perimount** ***n*** **= 2,630**	**Trifecta** ***n*** **= 2,423**	***p*** **-value**
Isolated AVR, *n* (%)	2,397 (81)	1,986 (82)	0.9
Concomitant procedures, *n* (%)	233 (19)	437 (18)	0.9
**Indication for AVR**
Aortic stenosis, *n* (%)	2,396 (91)	1,987 (82)	**<0.01**
Aortic insufficiency, *n* (%)	231 (9)	436 (18)	**<0.01**

*Significant values are bold*.

### Type of Prosthesis and Time of Surgery

**Figure 1:**
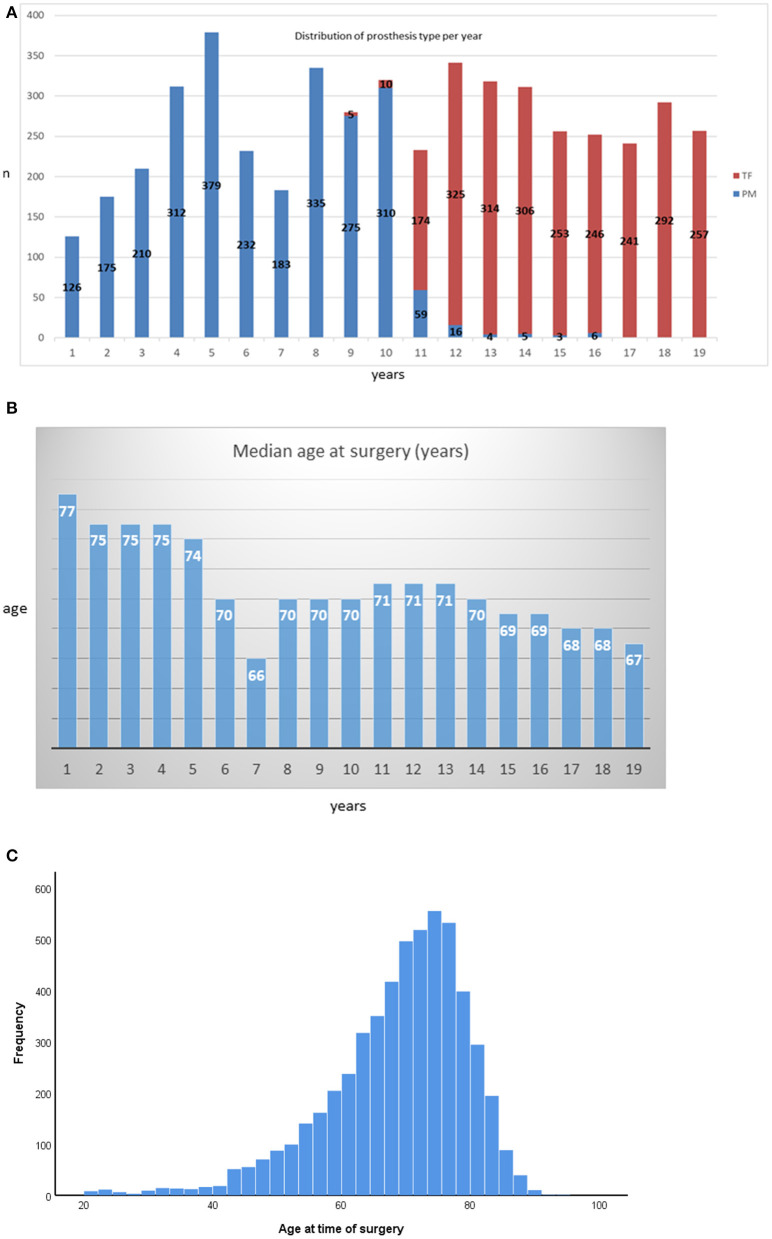
**(A)** Distribution of prosthesis type per year. **(B)** Annual distribution of median patient ages at surgery. **(C)** Histogram of patient ages at the time of surgery stratified by bioprosthesis type.

Perimount prostheses were predominantly implanted between 2001 and 2009, and Trifecta prostheses predominated between 2009 and 2019 (Figure 1A). The change in the praxis pattern was made based on institutional cost benefits. As such, the median follow-up in the Perimount group was 8 y [1.6 to 19 y; IQR = 3–10y], while that in the Trifecta group was 3.5 y [1 to 8 y, IQR = 0.3–3.4y]. The median patient age at time of surgery has decreased over time (Figure 1B). The distribution of patient ages stratified for prosthesis type is shown in Figure 1C. The distribution of the different prosthesis size is depicted in Supplementary Table.

### Mortality

**Figure 2:**
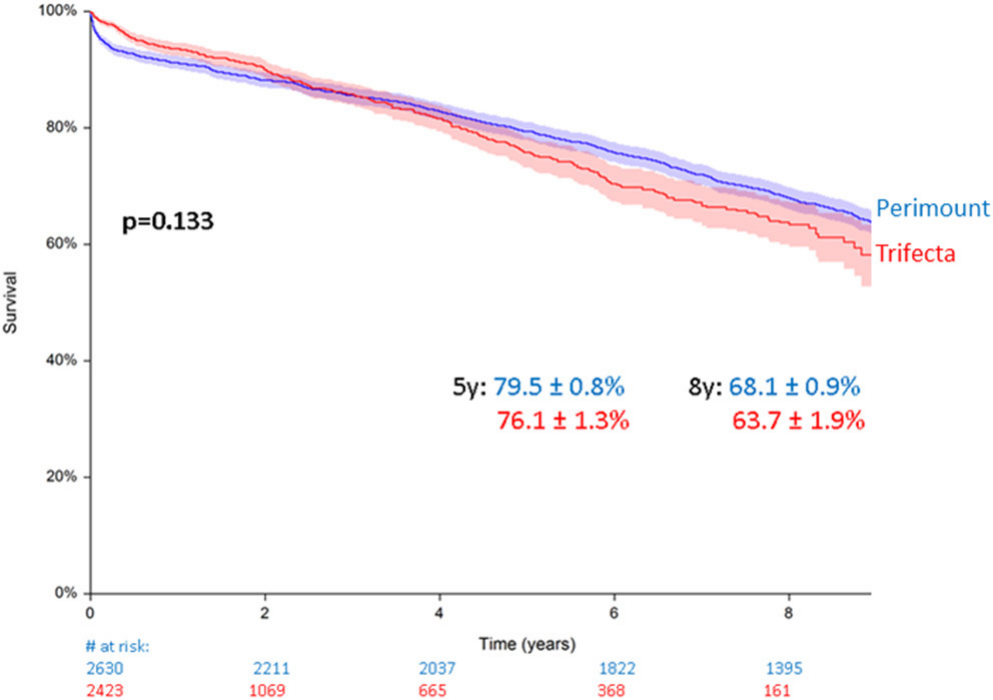
Survival after surgical aortic valve replacement (SAVR) using Perimount and Trifecta bioprostheses.

Total all-cause mortality in the Perimount group was 1,201 (45.6%) during a maximum follow-up of 19 years and 389 (16%) in the Trifecta group during a maximum follow-up of 8 years. Estimated 5- and 8-year survival were similar in the two groups. A clear trend was identified with worse survival rates for the Trifecta group, which did not reach statistical significance: 79.5 ± 0.8% in the Perimount group and 68.1 ± 0.9% in the Trifecta group at 5 years and 76.1 ± 1.3% (PM group) and 63.7 ± 1.9% (TF Group) at 8 years (*p* = 0.133, Figure 2).

### Reoperation for Bioprosthetic Valve Failure

A total of 309 (6.1%) patients (*n* = 187 PM vs. *n* = 122 TF) required repeat aortic valve replacement because of BVF. The median time to reoperation was 8 y [3 to 19 y] in the PM group and 4 y [0.6 to 8.5 y] for the TF group. Median patient age at time of redo was 67 y [24 to 92 y] in the Perimount and 71 y [25 to 86 y] in the Trifecta groups (*p* = 0.045). In terms of procedure type, 167 of 187 patients with BVF in the Perimount group (89%) had surgical valve replacement vs. 91/122 (74.5%) in the Trifecta group (*p* < 0.01), whereas 31/122 (25%) of Trifecta patients with BVF had transcatheter valve-in-valve procedures vs. 20/187 (10%) in the Perimount group (*p* < 0.007).

**Figure 3:**
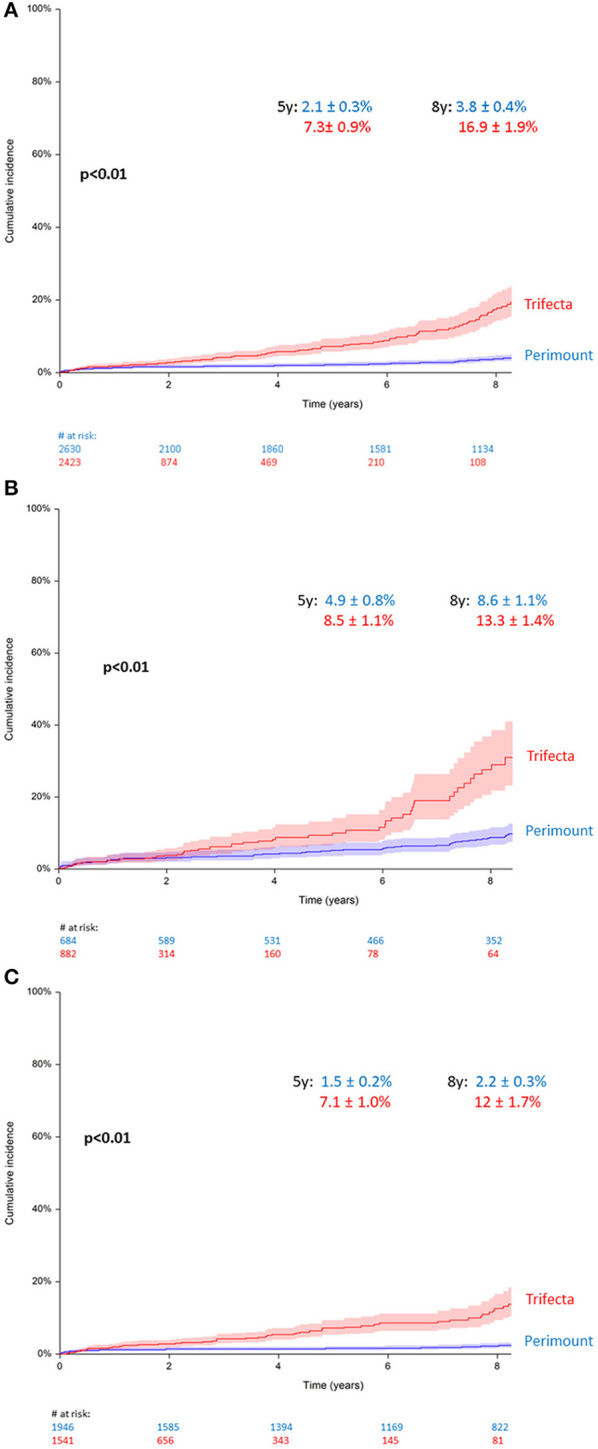
**(A)** Cumulative incidence of redo operation for bioprosthetic valve failure (BVF), all patients. **(B)** Cumulative incidence of redo for BVF in patients younger than 65 years. **(C)** Cumulative incidence of redo for BVF in patients older than 65 years.

At 5 years, the cumulative incidence of AV-reoperation was 2.1 ± 0.3% in the Perimount group and 7.3 ± 0.9% in the Trifecta group, and at 8 years, the cumulative reoperation rate in the Trifecta group was 16.9 ± 1.9 vs. 3.8 ± 0.4% in the Perimount group (Figure 3A), and this difference was significant (Grey's *p* < 0.01).

Upon sub-analysis including only patients younger than 65, the between-groups difference in the cumulative reoperation rate remained significant (*p* < 0.01), with a 5-year cumulative reoperation rate for BVF of 8.5 ± 1.1% in the Trifecta group and 4.9 ± 0.8% in the Perimount group, and an 8-year cumulative reoperation rate of 13.3 ± 1.4% (TF) vs. 8.6 ± 1.1% (PM) (Figure 3B). A second sub-analysis including only patients older than 65 showed similar results, and the cumulative reoperation rate for BVF remained significantly higher in the Trifecta group compared to the Perimount group (*p* < 0.01). Among those older than 65, the 5-year cumulative reoperation rate was 2.2 ± 0.3% in the Trifecta group vs. 1.5 ± 0.2% in the Perimount group and the 8-year cumulative reoperation rate was 12 ± 1.7% in the Trifecta group vs. 7.1 ± 1.0% in the Perimount group (Figure 3C).

### Composite Grafts

**Figure 4:**
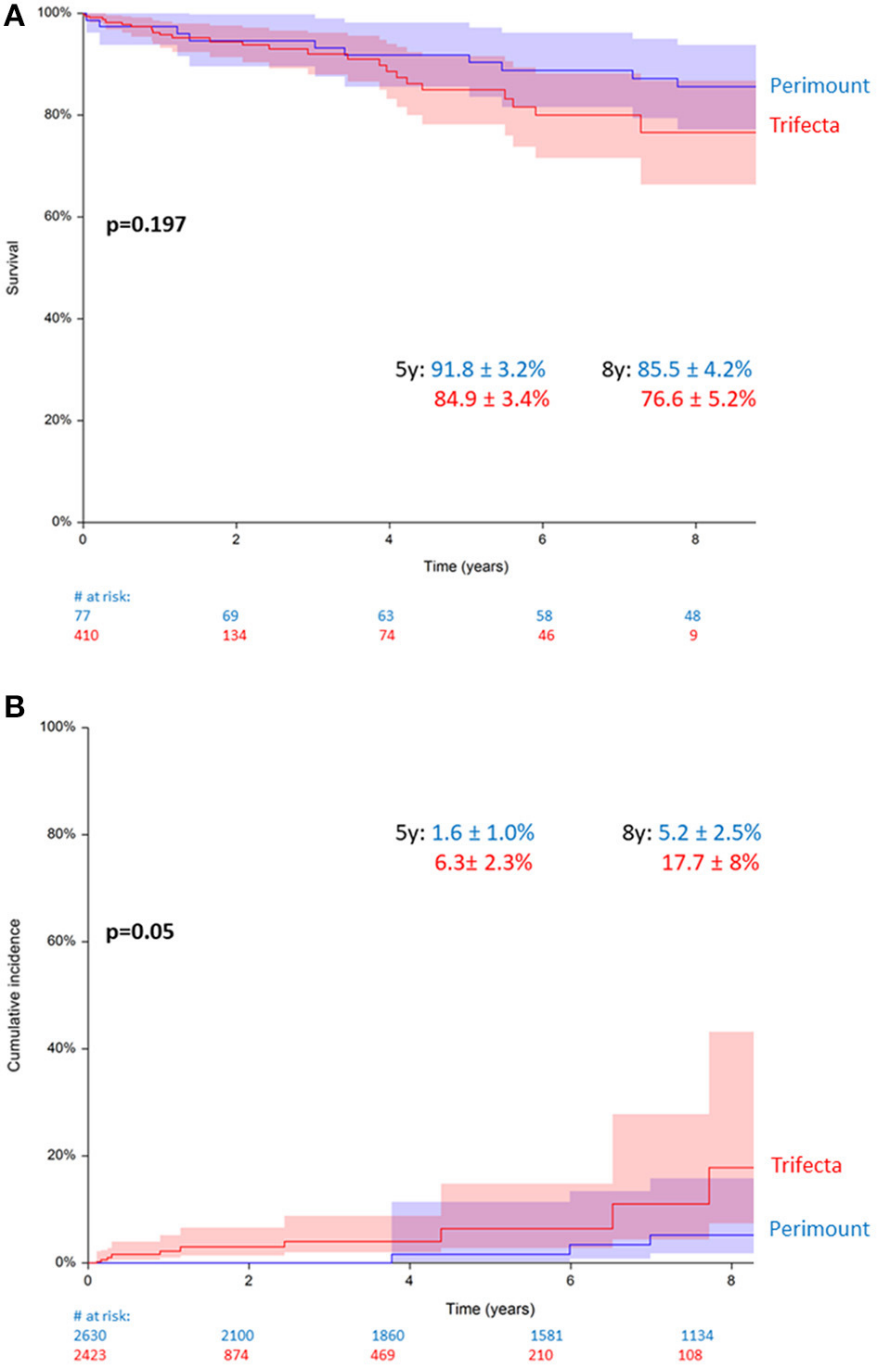
**(A)** Survival in patients with Perimount- and Trifecta-composite grafts. **(B)** Cumulative incidence of redo operation for bioprosthetic valve failure (BVF) in patients with Perimount- and Trifecta-composite grafts.

A total of 487 patients (*n* = 77, PM, vs. *n* = 410, TF) submitted to elective (non-ATAAD) aortic root procedures with a composite graft. Among these, the estimated 5- and 8-year patient survival rates were 91.8 ± 3.2% (PM) and 84.9 ± 3.4% (TF) and 85.5 ± 4.2% (PM) and 76.6 ± 5.2% (TF) (*p* = 0.197, Figure 4A). Among all patients with composite grafts, 35 had BVF requiring redo surgery (*n* = 9, PM, vs. *n* = 26, TF). The 5- and 8-year cumulative reoperation rates were 1.6 ± 1.0% (PM) and 6.3 ± 2.3% (TF) and 5.2 ± 2.5% and 17.7 ± 8% (*p* = 0.058, Figure 4B).

We did not find any significant differences in all endpoints comparing the different generations of both bioprostheses. We did not find also any significant differences in all endpoints comparing the different valve sizes.

## Discussion

### Unequal Redo Rates

The reoperation rate for BVF was significantly higher in the Trifecta group compared with the Perimount group (17% TF vs. 7% PM at 8 y, *p* < 0.01). Recent non-comparative studies have reported rates of early structural degeneration (5 years) in 11 to 13% of patients with implanted Trifecta valves (2–4). At this writing, the only other comparative investigation between Trifecta and Perimount prostheses is the recently published multicenter FinnValve registry-based study of 2,216 patients, which has shown similar results to ours, with a 6 vs. 0% reoperation rate at 7 years in patients with Trifecta and Perimount prostheses, respectively. The earlier degeneration of the Trifecta bioprosthesis in the FinnValve study was primarily observed in patients >65 years old, and only two patients were under 65 years of age (5). In our cohort, after subdividing our patients in two age groups (<65 y and >65 y), the reoperation rate remained significantly higher in the Trifecta group in both age groups (13% TF vs. 8.5% PM, *p* < 0.01, for patients <65 y, and 12% TF vs. 7% PM, *p* < 0.01, for patients > 65 y). We believe that nowadays with the improved quality of life and medical care in developed countries, the biological age is often not correlating with the chronological age. Patients which are older than 65 remain with almost same life activities compared to their younger opposers.

### Reasons for Reoperation

To date, the predominant finding upon reoperation in patients with BVF has been structural valve deterioration (SVD) with increased transvalvular gradients (4, 6, 7), and this finding was confirmed in the present investigation (6.8% TF vs. 0.6% PM at 8 years). Consequently, we ceased the use of Trifecta bioprosthesis for SAVR in our department. There may be two mechanisms responsible for the difference in the incidence of SVD between Perimount and Trifecta prostheses. First, the anticalcification agents for leaflet preservation in the Trifecta valve may play a significant role, and may have an impact on the accelerated SVD process (3, 4, 8). Next, the valve design, with externally (TF) or internally (PM) mounted leaflets, may contribute. Yankah et al. (9) reported on 1,500 patients with the Mitroflow-brand prosthesis, which has a similar design to the Trifecta prosthesis, with externally mounted leaflets, and found a significantly higher rate of SVD in younger patients (<65 years), although it remained unclear whether the design had an impact or if the finding was mainly related to the patient age. In the present study we found higher reoperation rates for the bioprosthesis with externally mounted leaflets (TF) independent of age. Thus, we may assume that the valve design may have an impact on the rate of degeneration. We assume that the leaflet mounting (internal vs. external) is not the only difference in these bioprostheses, and differencies in valve durability could be due to other factors such as anti-calcification processing and subtle aspects of design and production. Finally, in our opinion, the mechanism that leads to BVF is probably multifactorial and not only design-related.

Another reported cause of Trifecta valve failure that may in fact be design-related is cusp tear (4), which was the cause of BVF in 6 reoperations in our Trifecta group. All 6 patients presented with severe regurgitation (Supplementary Figure 1). Although tear on the non-coronary cusp was observed more often than on the other cusps, the exact mechanism warrants further clarification. Campisi et al. (10) have proposed that the cusp tear may be a result of mechanical damage during valve insertion and knot tying, and the next-generation Trifecta valve (Trifecta GT with glide technology) has a new holder with legs that are positioned in front of the leaflets for added protection during valve insertion and knot tying, and internal back-stops to protect the stent posts from deforming during valve insertion. However, the effect of this new design is unknown at this point. After the FinnValve study (5) reported significantly increased redo rates in patients with Trifecta prostheses, an invited commentary (11) argued that the high redo rates may be more related to the implant-and knot-technique exerting excessive pressure on the strut base than to the type of bioprosthetic design. Therefore, we performed a subgroup analysis of the 487 patients in our cohort who received composite grafts. In these patients, no manipulation of the Trifecta prothesis was required and thus, any mechanical damage to the Trifecta prothesis should be excluded. However, even in this subgroup, we found a clear tendency of higher redo rates in patients with Trifecta-composite grafts, which just failed to reach significance (*p* = 0.058). Assuming that the knot technique during the implantation of a composite graft is a no-touch technique in terms of the prosthesis strut base, our data do not support the hypothesis that the implantation technique may be the leading cause for the shorter durability of the Trifecta bioprosthesis.

### Bioprosthesis Type and Patient Survival

We designed this study excluding the patients with acute endocarditis and ATAAD, assuming that these two indications may exert an influence on patient long-term survival. Accordingly, the present analysis showed no difference in long-term survival between the Perimount and the Trifecta groups at 8 years (64% TF vs. 68% PM, *p* = 0.133). Our findings are in line with those from the Finn-Valve study, where at 7 years the all-cause mortality was reported to be 32% in patients with Trifecta vs. 23% in patients with Perimount (*p* = 0.755). To our knowledge, to date, there is only one comparative study between these prostheses, with very similar findings as ours (12). Yongue et al. (12) compared recently the durability and hemodynamic performance of 2,298 Trifecta prostheses with 1:1 propensity matched Perimount prostheses. In their study Trifecta bioprosthesis exhibited superior early hemodynamic performance, but had a rapid increase in transvalvular gradient and more aortic regurgitation, with lower freedom from explant at 5 years, which raises concern regarding long-term Trifecta durability despite favorable early hemodynamics (12).

In the past, different prosthesis with internally mounted (Ionescu-Schilley™, Soprano™) and externally mounted leaflets (Labcor dokimos™) have been used. Considering that the newest generation bioprosthesis with internally mounted leaflets-PM Magna Ease™ (Edwards), Inspiris Resilia™ and Avalus™ (Medtronic), as well as newest generation of those with externally mounted leaflets-Mitroflow™ (Sorin) and Trifecta GT™ (Abbott) will further be manufactured, future comparative and randomized studies could give accurate perspective in the future.

### Future Perspectives

Improvements in bioprosthetic durability and improved outcomes in re-operative aortic valve surgery, including the possibility of transcatheter valve-in-valve implantation, have led to the increased use of bioprosthetic aortic valves in younger patients. The transcatheter development and expansion nowadays, is influencing completely the SAVR trend and even the indication at baseline. In our cohort the indications for SAVR (aortic stenosis vs. regurgitation) was significantly different between Perimount and Trifecta groups. This could be a reflection of Perimount being used in the early part of the series and Trifecta in the late part, when there was more use of TAVR for aortic stenosis.

Although the number of transcatheter techniques is also growing and the TAVR-in-SAVR procedure seems to be technically attractive, there are still at least two important aspects that must be taken into account when choosing the type of bioprosthesis for initial SAVR: the risk of coronary obstruction and the possibility of valve cracking. Ribeiro et al. (13) reported on 1,612 patients in the Vivid-registry and found a significantly higher incidence of coronary obstruction during TAVR-in-SAVR in patients with bioprostheses with externally mounted pericardial leaflets compared to those with internally mounted leaflets (6.4 vs. 0.7%, *p* < 0.01). The factors that may favor coronary obstruction seem obvious, as the distance from the leaflet to the sinus wall is shorter and the leaflets are higher. Furthermore, the titanium frame of the Trifecta valve does not allow later fracture to increase the bioprosthetic valve ring in order to implant a larger transcatheter valve (14).

## Conclusion

The present comparative study shows higher rates of reoperation due to BVF among 2,423 patients undergoing SAVR with the Trifecta bioprosthesis with externally mounted leaflets compared to 2,630 patients with a Perimount valve with internally mounted leaflets. BVF risk was not influenced by age, and patients in both groups had similar long-term survival.

## Limitations

This study is limited by its retrospective and non-randomized single-center design. The available echocardiographic reports were performed in a variety of clinical settings, and the definition of SVD varied among these readings. Furthermore, causes of death were not specified in our cohort, and mortality rates were analyzed as all-cause and there is a lack of covariate-adjusted analyses. In this circumstance, patients who died from sudden death, due to inoperability, or with undiagnosed severe bioprosthetic valve failure, have not been considered in the analysis.

## Data Availability Statement

The raw data supporting the conclusions of this article will be made available by the authors, without undue reservation.

## Ethics Statement

The study was approved by the Institutional Review Board of the Technical University of Munich (129/21 S from March/5/2021). The approval included a waiver of informed patient consent.

## Author Contributions

KV and RL: conceptualization and project administration. KV and ZA: methodology. KV: software, formal analysis, and supervision. KV, RL, and MK: validation. ZA: investigation, resources, and data curation. RL, ZA, and KV: writing—original draft preparation. JB, MK, and SV: writing—review and editing. RL: visualization. All authors have read and agreed to the published version of the manuscript.

## Conflict of Interest

The authors declare that the research was conducted in the absence of any commercial or financial relationships that could be construed as a potential conflict of interest.

## Publisher's Note

All claims expressed in this article are solely those of the authors and do not necessarily represent those of their affiliated organizations, or those of the publisher, the editors and the reviewers. Any product that may be evaluated in this article, or claim that may be made by its manufacturer, is not guaranteed or endorsed by the publisher.

## Abbreviations

SAVR: surgical aortic valve replacement
PM: Perimount
TF: Trifecta
BVF: biological valve failure
ATAAD: acute type A aortic dissection
HR: hazard ratio
CI: confidence interval
COPD: chronic obstructive pulmonary disease
PHT: pulmonary hypertension
SVD: structural valve deterioration
TAVR: transcatheter aortic valve replacement.
