# Supplementary figures and images for: Different Rates of Bioprosthetic Aortic Valve Failure With Perimount™ and Trifecta™ Bioprostheses
Source: Front Cardiovasc Med. 2022 Jan 20;8:822893. doi: 10.3389/fcvm.2021.822893 (PMC8811120; doi:10.3389/fcvm.2021.822893)

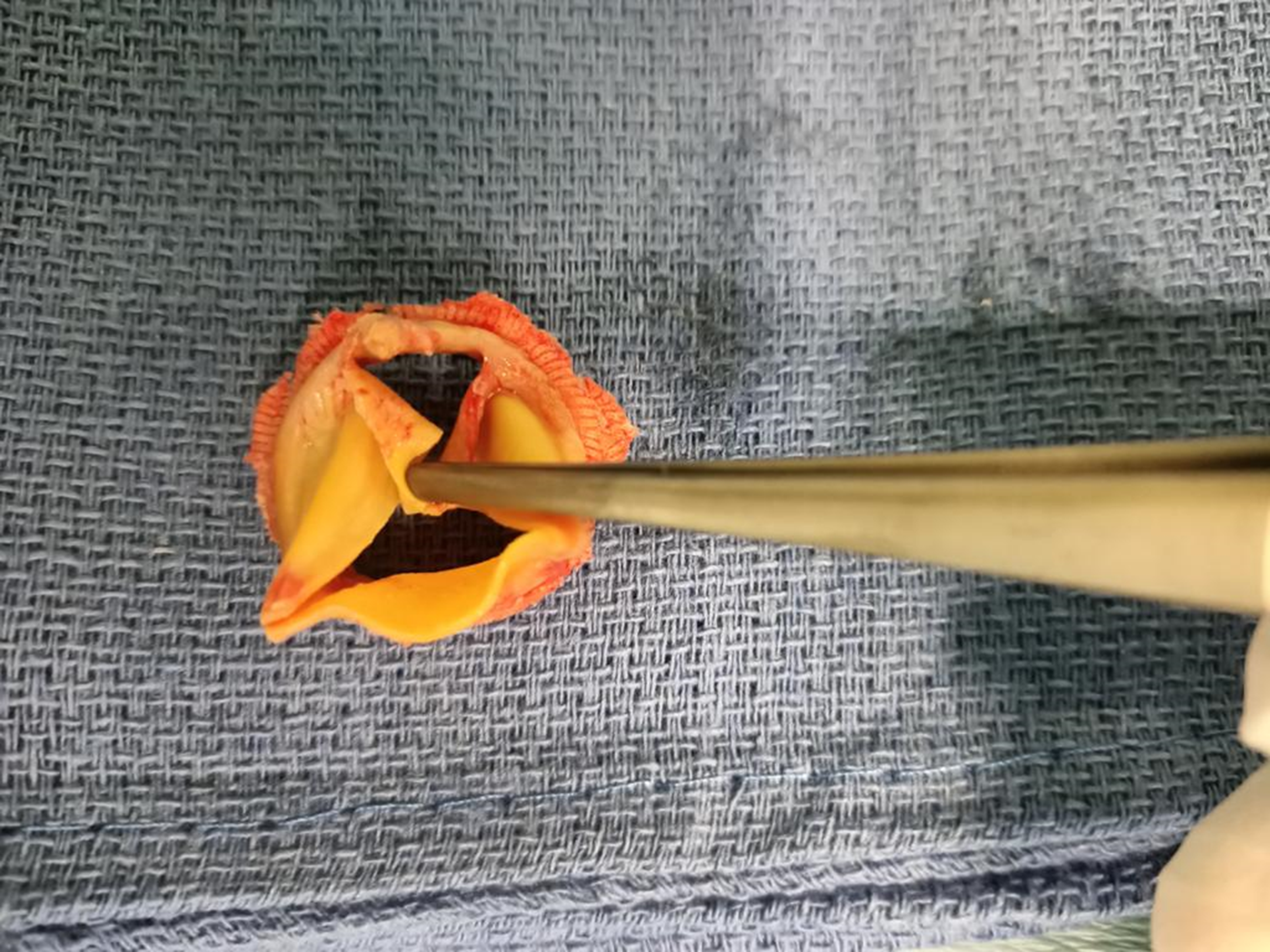

Supplement: Supplementary file 1 [file Image_1.TIF]

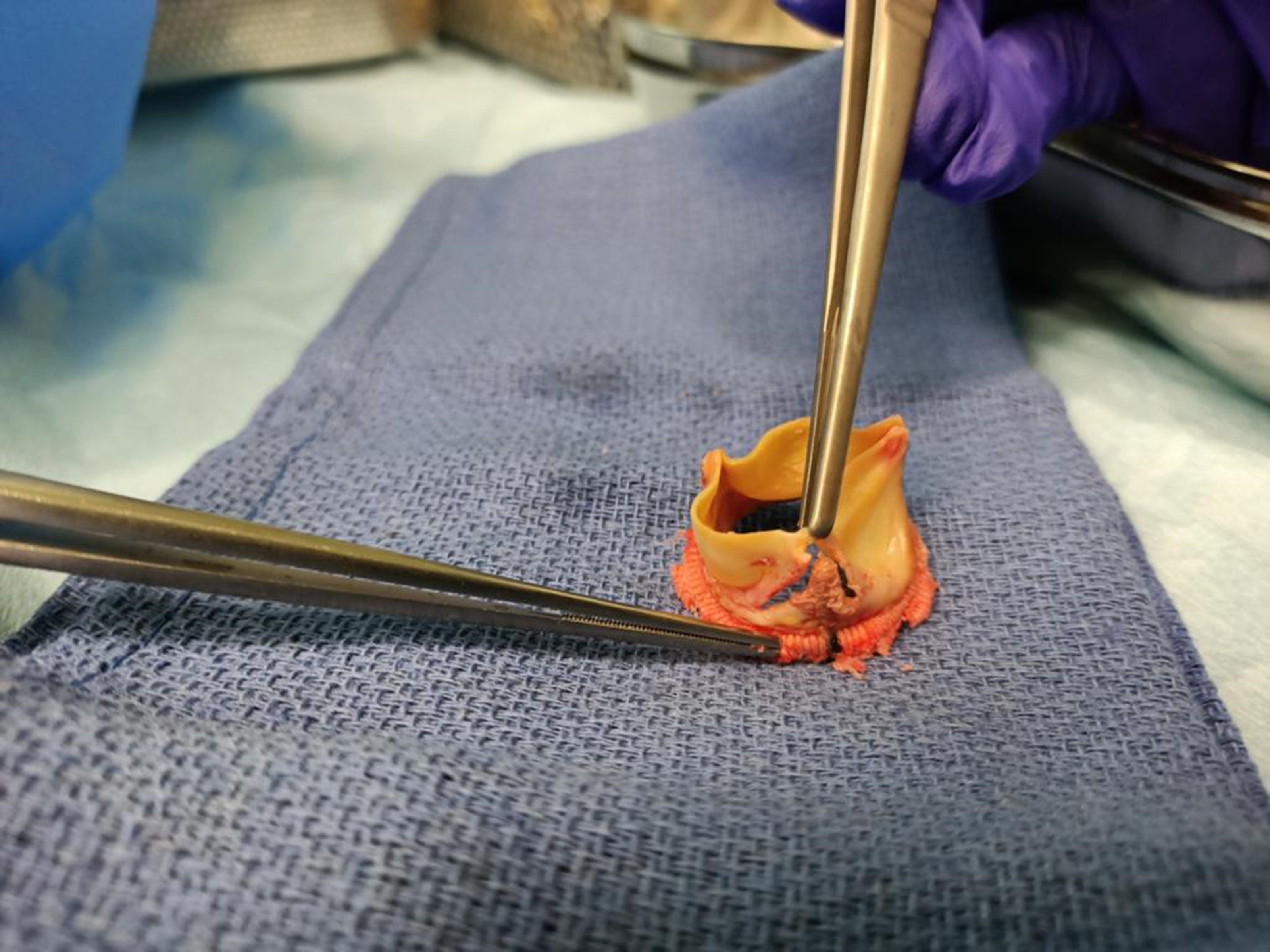

Supplement: Supplementary file 2 [file Image_2.TIF]
