# Supplementary material for: Different Rates of Bioprosthetic Aortic Valve Failure With Perimount™ and Trifecta™ Bioprostheses
Source: Front Cardiovasc Med. 2022 Jan 20;8:822893. doi: 10.3389/fcvm.2021.822893 (PMC8811120; doi:10.3389/fcvm.2021.822893)
Supplement: Supplementary file 3 [file Table_1.pdf]

Supplementary Table
Distribution of prosthesis size

Prosthesis size	Perimount, n=2630	Trifecta, n=2423
19, n (%)	116 (4.4)	80 (3.3)
21, n (%)	716 (27.2)	506 (20.8)
23, n (%)	964 (36.6)	636 (26.2)
25, n (%)	542 (20.6)	603 (24.8)
27, n (%)	228 (8.6)	393 (16.2)
29, n (%)	64 (2.4)	205 (8.4)
